# Co-Creation Hub Is the First Step for the Successful Creation of a Unified Urban Ecosystem-Kaunas City Example

**DOI:** 10.3390/ijerph19052609

**Published:** 2022-02-24

**Authors:** Akvilė Feiferytė-Skirienė, Lina Draudvilienė, Žaneta Stasiškienė, Sergej Sosunkevič, Kastytis Pamakštys, Laura Daniusevičiūtė-Brazaitė, Inga Gurauskienė

**Affiliations:** 1 Institute of Environmental Engineering, Kaunas University of Technology, Gedimino St. 50, 44239 Kaunas, Lithuania; lina.draudviliene@ktu.lt (L.D.); zaneta.stasiskiene@ktu.lt (Ž.S.); kastytis.pamakstys@ktu.lt (K.P.); inga.gurauskiene@ktu.lt (I.G.); 2 Department of Medical Technology and Dietetics, Kaunas University of Applied Sciences, Muitines St. 15, 44280 Kaunas, Lithuania; doctor@inbox.lt; 3 Faculty of Social Science, Arts and Humanities, Kaunas University of Technology, K. Donelaicio St. 73, 44029 Kaunas, Lithuania; laura.daniuseviciute@ktu.lt

**Keywords:** urbanization, unified ecosystem, coordination center, sustainably and healthy life, national government, city public authorities

## Abstract

The identification of the main steps for the creation of a unified ecosystem from the institutional point of view and the framework for ecosystem design is presented and discussed. Based on the expertise and the knowledge gained during the time when the ELISE project had been implemented, a unified Kaunas city ecosystem is being designed using the Ecosystem Map method. As the review of the ELISE project reports helped to identify the main steps of each project partner in building ecosystems’ networks, Kaunas city chose to create a co-Creation Hub (c-CH), which is the first step in developing an ecosystem management model. The main tasks of such a hub are listed, and should involve the preparation of a long-term action plan involving not only the coordination of the stakeholder meetings, organisation of seminars, the preparation of new materials, and methodology but also the development of a clear strategy for each stakeholder based on national economy and government and municipality policies. The role of the c-CH is to ensure the ease of cooperation and knowledge distribution among stakeholders within the city, public authorities, and the national government. This approach could become a fundamental background tool for the regional and/or city municipal and stakeholder-based creation and development of unified ecosystem development.

## 1. Introduction

The enormous scale and rapid pace of urbanisation has led to the growth of cities and a concentration of people in them. As a result, a new world name, the “planet of cities” has been introduced [1]. All the regions of the world with strong economies are faced with an increasing population residing in cities as a result of internal and external migration. This is confirmed by calculations made and future forecasts [2]. According to the United Nations data presented in the “World urbanisation prospect 2018” [3], more than a half of the world’s population, 55.3%, lives in urban areas. In Europe where more than 80,000 cities and towns can be counted, this number is 74.5%. [4]. By 2050, 68.4% of the world’s population is projected to be living in urban areas, with 83.7% of Europe’s population doing the same, making urbanisation one of the most transformative trends of the century. The urban population increased from 29.6% to 53.9% from 1950 to 2015, respectively [3,5]. The urbanisation drift is accelerating across the world, bringing a grand and new challenge with it, as rapid urbanisation transforms the social, political and economic face of the world [6,7,8]. In addition, the growing concentration of cities, together with the population and the growth of new large cities, causes environmental and ecological changes [8,9,10,11,12] which directly affect the living environment, and public health [13]. Despite the fact that urban living offers a lot of economic, social, technological, and educational opportunities [14], it is associated with higher levels of air pollution, toxins, traffic noise, temperature changes, and poorer water and food quality, which is affecting the well-being and quality of life of citizens [15,16,17,18,19,20]. Therefore, a great number of discussions and studies have been undertaken to understand the changes of the physical and mental health of urban populations as cities evolve [20,21,22]. Thus, the identified challenges make us think not only about processes that create and support urbanisation, but, at the same time, they make us conceive of processes that ensure the functionality and sustainability of the city [23,24,25,26].

Unlike natural ecosystems, cities contain a number of different ecosystem components that are mostly human-constructed and involve a broad range of microenvironments and biological assemblages, from natural ecosystems and human built environments [27]. The unified understanding and administration of ecosystem services, taking into consideration social, geographical, urban differences in the city are important to ensure social justice and equal access to ecosystem services and goods for all citizens. Ecosystem services in urban areas differ from ecosystem services in other areas since they include non-ecological elements, such as human-built infrastructure, technology, social and economic services [28,29]. The concept of ecosystem services suggests the provision of goods and services for humans. However, they can be achieved when the demand for them is realised [30]. To address the issue of how ecosystems in cities can contribute to sustainability issues and promote well-being, human and non-human stakeholder interaction and possible cooperation frameworks in the generation of urban ecosystem services needs further scientific investigation [31,32]. A multidisciplinary and multi-sectoral approach among researchers and practitioners from diverse disciplines such as psychology, sociology, public health, clinical medicine, technology, marketing, business, organisational behavior, communications and other fields, is necessary to plan, implement and assess ecosystem creation. Therefore, cooperation among different named factors is easier on the regional and/or city-level [33]. However, in order to bring together all sectors in the region and/or city, the coordination challenge should be solved first.

The ELISE project shows that the specific political tasks related to social economy, such as economic growth, employment, financial returns and wealth creation, need to be solved when an ecosystem is being built. However, given the multidisciplinary and interdisciplinary nature of the ecosystem and the huge number of participants, it requires constant coordination and maintenance.

The aim of this research is to identify the main steps for the creation of a unified urban ecosystem and to provide its design framework following the ELISE project findings; to present an institutional coordination and communication centre as a first step, with certain tasks and functions, in creating a unified urban ecosystem., to identify and explore the challenges that such a centre should solve; and to provide opportunities for change by raising awareness, building capacity, fostering research, practicing collaboration and connecting all citizens.

## 2. ELISE Project

The INTERREG European Life Science Ecosystems (ELISE) project involves seven different geographical and economic profiles of European regions so as to find ways to promote better health and well-being for all, including economy-related benefits such as market growth and job creation, along with a reduced burden of health problems on individuals, health and care systems, and society. The longer-term goal of the ELISE project is focused on green energy, sustainable environment, a circular economy to improve human well-being based on cooperation between research and development, and innovation for the creation of Life Science Ecosystems. These are the main goals, which can be implemented with ELISE assistance. The literature analysis results confirm a research gap, with a need for a multidisciplinary and multi-sectoral approach, with researchers and practitioners working together to plan, implement, and evaluate. The main aspects of the ELISE project that could cover and help solve the named tasks are as follows:

- The creation of new information and communication systems to support ecosystem infrastructures.
- The transfer of knowledge between science, business, and community.
- The transfer of technology between science and business.
- Regional cooperation focused on promoting innovation to solve medical needs of ageing populations.

Based on the ELISE project and the European Commission recommendations, the creation of an ecosystem is advised to begin at the regional and/or a city level [33]. The unified urban ecosystem should be sustainable and comprise an innovative structure that uses all means, and links all participants by improving quality of life, ensuring the efficiency of urban operation and services, and competitiveness [34]. Such a type of ecosystem would involve the unified cooperation of all sectors existent in the region where the ecosystem is being built [35], because the approach to the systems involved is very important for the creation of a unified urban ecosystem [36].

## 3. Materials and Methods

### 3.1. Sustainable Development Goals and Ecosystems Creation

A dedicated SDG 11 focuses on cities and human settlements and requires consolidated actions and partnership across various stakeholders on international, national and local levels, whereby national and international bodies and government, education, public and private sectors’ actors work together [37,38,39]. Maes et al. [40] found that 91 targets (54%) of the 2030 Agenda are related to urban ecosystems, including protection, assurance of equal rights to different type of services and management through multilevel governance frameworks. It confirms the importance of urban ecosystems in the efficient and successful sustainability transition.

Cities with a high concentration of innovation, knowledge, culture, and financial and non-financial sources are able to pursue better social and economic opportunities and are the key drivers of global urbanisation trends [40]. Cities are the best places to make connections between people because of the existing physical and social infrastructure. Cities unlock and empower economic, human, and social changes with an integrated sustainable policy [41]. The importance of cities in sustainable development is recognised in the New Urban Agenda [5] in which a framework provides how to achieve SDGs and promotes cities to act as hubs that seek balanced, sustainable and integrated territorial development, promoting their ecosystems through sustainable and inclusive urban economies.

The creation of better cities that are more sustainable, equitable, democratic, and productive would help to address many of the salient challenges [1]. With regard to these issues, solutions are being sought globally. One method by which to do this is through the creation of a ecosystem in urban areas which has direct implications on the environment, economy, society, and politics of a region and country [42,43,44], while at the same time involving all the manifestations of human life and influencing human health, well-being and lifestyle [45]. Therefore, it is stated that human health is a part of ecosystem health [35]. So, an interest in the impacts of urban ecosystem on human health and well-being is growing continuously [46,47,48], and the creation and development of the various types of ecosystems has been a topic of importance in recent years across the world [49]. Maes et al. [40] studied the concepts of welfare and well-being manifested through peaceful, transparent and accountable institutions (SDG 16) and governance, narrowing gender gaps (SDG 5), and addressing health challenges (SDG 3). I. Douglas [47] associates human well-being with urban greenspaces, vegetated areas, water bodies and, how the characteristic of urban landscape design affects human health. J. Munksgaard et al. [50] present urban ecosystems, human health and well-being as a transboundary environmental trade whereby the consumption and behaviour of one urban area can have an impact on the health and well-being of people in another area. The diversity of conditions in urban areas creates different factors that have an impact on citizens’ health and well-being. These factors can be divided into four risks groups as follows: environmental (air quality, noise, soil and water contamination, waste disposal) economic (affordable housing, access to services, income level, customer purchase power), social and individual (crime, violence, inequality, social exclusion), technological (traffic incidents, industrial and chemical disasters, soil and water contamination from mass production) risk factors [47]. All these disruptions in ecosystems are likely to impact humans’ well-being and ecosystem functions [51].

### 3.2. Research Design

**Figure 1:**
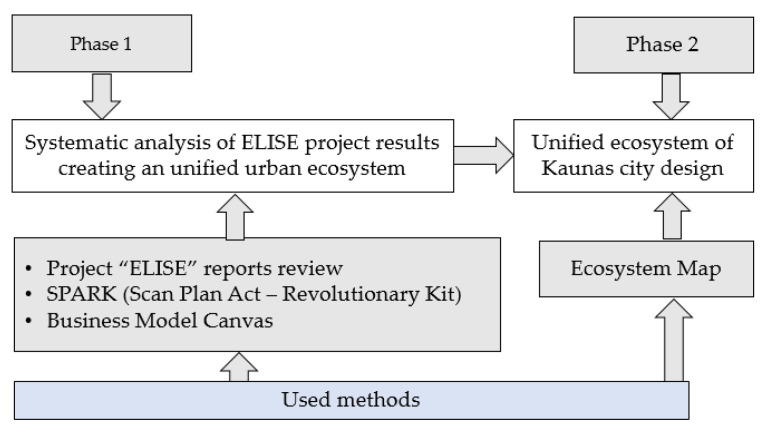
Methodology.

This study was designed to map urban ecosystem components based on the ELISE project outcomes and Kaunas project by using the c-CH as an ecosystem model. The research conducted and the methodology used are presented in Figure 1.

The transition to sustainable urban development and the creation of unified urban ecosystems requires innovative business models, products, and services. The Business Model Canvas was developed by Osterwalder and Pigneur [52] as a tool that can be used in business growth, providing a complete overview of how the current service/product model could be improved. The Business Model Canvas was used to create a context in which researchers can measure the real feasibility of research activities with a marketable perspective. It brings together researchers, businesses, civil society, end-users, innovation centres, policymakers, and development agencies in order to identify and discuss research project elements, train researchers to present their ideas and then put together mixed teams of these stakeholders to work together on the particular Business Model Canvas.

SPARK (Scan Plan Act—Revolutionary Kit) is a tool used to facilitate the dialogue and collaboration between companies, civil society, end-users, policy makers and industrial-research laboratories. It helps researchers to understand research results and outcomes from a market point of view. The SPARK tool helps to improve the marketability of research-based products and services, and enhances the synergy between stakeholders and innovation ecosystems.

The ELISE project began in 2017 and lasted until the end of 2021. Meetings with partners were organised and the reports of these meetings were published on the INTERREG Europe website every 6 months. While the first reports provide an overview about the project, the initial steps and plan building activities, the latest reports provides storytelling of each project partner and their results while building ecosystem networks. A review of the ELISE project reports allowed us to identify the main steps of each project partner when building ecosystem networks and present different ecosystem network design frameworks for beginners and advanced level. As a result of the ELISE project, the Ecosystem network design framework was constructed using information obtained from the ELISE project reports review and a Unified Kaunas city ecosystem c-CH is being designed, using the Ecosystem Map method to identify all stakeholders, flows and relationships based on the expertise and gained knowledge during the time of the ELISE project implementation. The main objective of such an ecosystem is to ensure the fluid cooperation and knowledge distribution between different stakeholders and the improvement of healthcare and well-being in Kaunas.

The SPARK tool was used in ELISE project partners’ meetings, sharing examples of good practices and during the site visits in Kaunas (Lithuania), Le Studium (France), Board of Trustees on Health Economy in Mecklenburg-Vorpommern, Germany and WellnessValley initiative created in Romagna, Italy. In the period from 2017 to 2020, project partners had 17 meetings that were held in different sites by stakeholders. Since March 2019, all project partners’ meetings have been held via MS Teams due to implemented COVID-19 travel restrictions.

### 3.3. Ecosystem Design Framework Based on ELISE Project Outcomes

**Figure 2:**
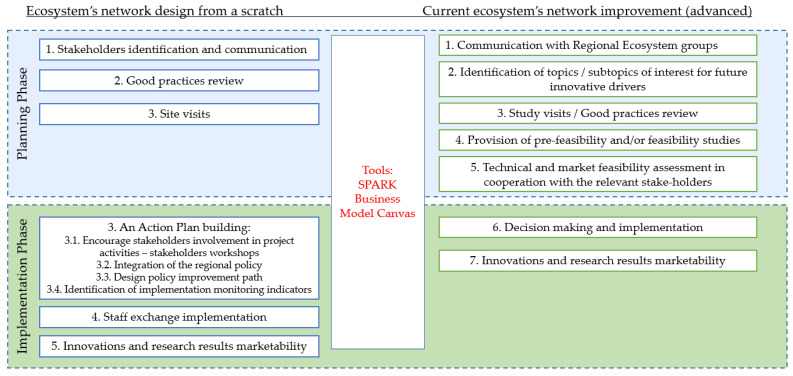
Ecosystems network design framework (prepared by authors).

The ELISE project facilitated the identification of states for ecosystem creation. Steps differ depending on the maturity of the region and existing fragments or maturity of the ecosystem’s infrastructure. The project partners with existing advanced ecosystem networks are Germany, France, Poland, the Emilia-Romagna region (Italy) and two partners without an existing ecosystem network—Lithuania and Slovakia. The ELISE project led to a better understanding of how ecosystem network implementation should be maintained, keeping in mind different conditions, experiences, and interests between the project’s partners. A summary of the identified types of ecosystem creation is presented in Figure 2.

*Ecosystem network design from scratch.* Stakeholder-identification and involvement in the ecosystem design process helps to identify strengths and weaknesses, good practices to be imported and make decisions on the next steps to be undertaken to make the most out of interregional learning activities. A list of the identified local stakeholders includes city’s public authorities at local level, representatives from national government (e.g., ministry of economics, ministry of environment), science institutions, business enterprises, public, and non-governmental institutions, etc.). The SPARK tool was used when fostering dialogue between Enterprise and Research by empowering researchers to think about research results from a market point of view on the basis of the Business Model Canvas.

At the regional level, the Lublin Marshall Office could be a good example of a developed methodology used to work with stakeholders effectively. Work with stakeholders was conducted following a four step approach as follows: (1) the individual work of an external expert, experienced in the Life Science topic, who proposed an original list of limitations of the Life Science ecosystem in the region; (2) discussion of the results was presented in the Regional Ecosystem Group’s (REG) meeting and workshop; (3) preparation of stakeholders’ feedback report; (4) based on the expert analysis, the report from the REG meeting and their own experience, the project team pointed out the most significant constraints.

Site visits (startups accelerators, incubators) can help to share the experiences of different ecosystems’ players or stakeholders, creating and developing future innovations, exchanging interregional practices, and improving inter-regional ecosystem cooperation. The study visits could be considered a great success by all stakeholders, both those with advanced Life Science Ecosystems and those who are working on developing new ecosystems in their regions. It is an excellent opportunity to gain an insight into a cluster’s activities, to identify motivation and barriers, to learn about their strategies, visit their facilities and see their good practices in action. Information and experiences present a good starting point for the development of the Ecosystem concept and components.

Ecosystem creation is based on four constraints included in the Action Plan, namely market, human resources, technical resources and bioethical concerns. Such an endeavor has to be addressed in relation to a legal framework, infrastructures and equipment, skills and competences, polices, and social environment. Successful policy instruments and actions supported by Public Administration and private initiatives related to the life science ecosystem can provide inspiration to other ecosystem partners. The Action Plan has to include actions for local and regional-level policy improvement to create and strengthen the Ecosystem in question. The Action Plan needs to identify indicators for each of the four constraints to ensure monitoring and successful implementation.

Staff-exchange implementation is another step in ensuring more efficient ecosystem implementation and experience transition between different stakeholders and separate ecosystems on national and regional levels. Each ecosystem works in a different environment, focusing on different areas, so stakeholders’ experience, interests and engagement can differ.

Innovation and results marketability is the final result of a created ecosystem, whereby different stakeholders interact to accelerate ecosystem’s services and innovations in different sectors.

*Current ecosystem’s network improvement (advanced)*. More advanced ecosystems, e.g., the Emilia-Romagna region, with existing ecosystem infrastructure, can choose a different approach, focusing on the methodology for the promotion of strategic projects with the full involvement of the regional “triple helix” that includes research laboratories, companies and public decision makers. The methodology begins with the identification of topics/subtopics of interest for future innovative drivers (strategic projects) matching the Regional needs. These strategic projects have to be able to engage the largest number of stakeholders, committing them to the finalization of the project and aiming to positively impact the innovative sub-topic in which they are engaged. This includes the provision of pre-feasibility and/or feasibility studies to help shape the project and to support the quest for strategic results. Finally, the process of a technical and market feasibility assessment in cooperation with the relevant stakeholders should be a continuum in order to provide the necessary support to decision making.

An effective coordination of the ecosystem’s activities can be of great value for each stakeholder and the surrounding society in achieving their respective objectives. The benefits include improved quality of life, well-being, more developed products and services with greater value-creation to the market through collaboration between business and research institutions.

## 4. Results

### 4.1. The Unified Ecosystem of Kaunas City

The strategic goal of the Lithuanian RIS3 is to create innovative technologies, products, processes and methods that respond to global and national challenges and to increase competitiveness (including commercialization of knowledge), thanks to the synergy between science and businesses, economic entities and other public and private sectors entities. As cities have recently been facing increasing environmental, social and economic challenges [35,53], based on the objectives of the ELISE project and the strategic goal of Lithuania, a unified ecosystem of Kaunas city is being created.

Kaunas is located in the middle of Lithuania, the area of which is 157 km^2^ (61 sq mi) and it is the second-largest city in Lithuania. Due to its favorable geographical location, Kaunas county is the only one in the Baltic states region in which two European transport corridors intersect, namely the I and IX B road corridors and I and IX D rail corridors and it makes for a favorable location for logistic business. It had a population of about 293 thousand at the beginning of 2020, which is 10.6% of the total population of Lithuania and the population density is 1.865 inhabitants/km^2^ [54]. It is an important centre of Lithuanian economic, academic, and cultural life. There are seven universities and four academies; therefore, Kaunas is often referred to as a city of students with about fifty- thousand students enrolled. With the development of the ELISE project, the idea is to develop Kaunas as a smart, environmentally friendly, innovative, clean, sustainable and green city which could be attractive for foreign investment. There is great potential to increase the quality of life in cities via the use of locally generated services [55]. Therefore, in order to create a sustainable urban ecosystem in which business, policy makers, academia, investors and citizens are ready to participate in joint projects, a framework for the integration and cooperation of the local city must be created and developed, bringing together all the named sectors.

### 4.2. Co-Creation Hub

Sustainable cities are living urban ecosystems that connect natural and artificial components which are strongly interconnected in sustainability design [56,57,58] and are integrated into a larger social organism [56]. The main aim of the urban ecosystem is to improve resilience and quality of life in cities [59] along with preserving natural resources. An ecosystem is a network of relationships among all actors, since “the performance of the city ecosystem does not depend only on physical capital, such as physical infrastructures, but also on human and social capital represented by the availability and quality of the knowledge, communication and social infrastructure” [60,61]. Therefore, for the creation of an urban ecosystem, the city needs to develop their own city’s vision, to define the strategies, and to engage with and mobilize all actors to participate in the new initiatives [62]. The participation and cooperation of the various urban actors can be made possible by the development of communication infrastructure that ensures the interaction of all actors [60]. Based on the named conditions, as a first step in creating a unified urban ecosystem, the creation of a center which could ensure the communication, cooperation and interrelation among all actors of the city should be established.

**Figure 3:**
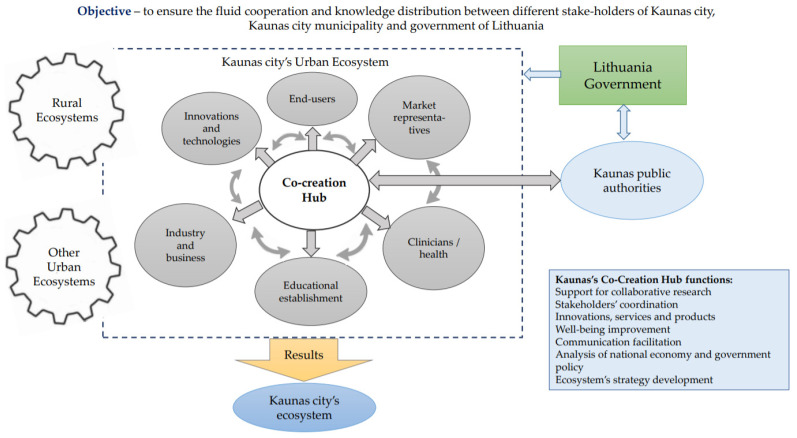
The model for the creation of the sustainable and unified ecosystem of the Kaunas city (prepared by authors).

Therefore, the beginning of the creation of a unified ecosystem of Kaunas city could be the creation of a new center of communication and cooperation. To this end, the new c-CH should initially address awareness-raising, capacity-building and collaboration through research and practice, creating new lines of communication between different members of the ecosystem (see Figure 3). Meetings with project partners and workshops helped to identify the main aim and functions of the c-CH using the SPARK tool. The main objective of such c-CH will be to ensure fluid cooperation and knowledge distribution between different stakeholders of Kaunas city, Kaunas city municipality and government of Lithuania. The main stakeholders of Kaunas c-CH are Kaunas City Municipality, The Kaunas City Municipality Public Health Bureau, Kaunas Universities—Lithuanian Sport University and Lithuanian University of Health Sciences. The creation process of Kaunas c-CH was based on connecting different participants and stimulating collaboration opportunities among them to create the Kaunas city ecosystem.

Workshops with Kaunas city’s stakeholders using the SPARK tool garnered a better understanding of the current political situation, different stakeholders needs and the possible outcomes for Kaunas city’s ecosystem after the c-CH’s implementation. The political situation in Lithuania and the laws adapted depend on the government of the country which directly affects everyone living in that country and also all political components in different regions and cities. The local authorities are directly responsible for the development and expansion of their regions and cities [55]. Therefore, the government of the country together with all local political components such as the authorities, council and mayor have a direct influence on the creation and development of the region and/or city. According to these factors, the c-CH should become a fundamental background among the region and/or city municipality and stakeholders involved in the unified ecosystem creating and developing. Due to this reason, the creation of a regional and/or an urban ecosystem requires:

- Direct cooperation of local authorities with the national government.
- Direct cooperation of local authorities with the c-CH.

As interdisciplinary cooperation in research and practice needs to be established and developed in order to support local authorities [49], this task could be conducted by the c-CH. The c-CH should help strengthen research, technological development and innovation, and improve an operational program for investing in the European Structural and Investment funds which is provided in the Strategic Plan of the Kaunas City Municipality. The c-CH should constantly perform a joint analysis of the national economy and government policy making, as well as of the municipal strategy of the Kaunas city. The target meetings would aim at developing a clear strategy for each stakeholder based on the national economy and policies of the government and Kaunas city municipality. It is also important that a carefully prepared action plan be formed on the basis of the work, and it ought to be planned for the long term. Only then will it be possible to implement strategic goals in practice. If all these conditions are met, such an instrument can act as an excellent prerequisite for the implementation of strategic objectives of a region. It is of great importance that a balance of the stakeholders in the target meetings is ensured. In particular, the participation of industry representatives plays a substantial role in ensuring that the needs of the economy are met, thus creating added value in the region. Thus, based on the identified work and tasks to be completed by the c-CH for communication and cooperation, it can be argued that it plays a key role in the creation, coordination and maintenance of a unified ecosystem.

## 5. Discussion and Conclusions

This study identifies the main steps for the creation of a unified ecosystem from the institutional point of view and presents and discusses a framework for ecosystem design. Based on the systematic literature review on ecosystems and a review of the ELISE project reports, we identify the main steps of each project partner in building ecosystem networks. This information allowed us to identify the different ecosystem network design frameworks for beginners and for a more advanced level. Taking this into account, the city of Kaunas chose to create a c-CH as the first step in developing an ecosystem management model. This approach could become a fundamental background tool among the region and/or a city municipality and stakeholders involved in unified ecosystem creation and development. Interdisciplinary cooperation in research and practice needs to be established and developed in order to support local authorities. This task should be conducted by a c-CH. Therefore, the main goal of the hub should be to ensure the fluid cooperation and knowledge distribution among different city stakeholders, public authorities, and the national government. The main contributions of such a c-CH is the coordination of stakeholder meetings, organisation of seminars, preparation of new materials and methods and the submission of new projects together with the authorities of the city to the national government. The hub should also constantly conduct a joint analysis of the national economy and government policy and the city municipality strategy. Accordingly, the c-CH can help to develop a clear strategy for each stakeholder, based on the national economy and the government and locals politicians. Despite the identified positive contribution of the c-CH to urban ecosystem and city’s well-being, challenges on the practical side might arise when the interests of one or few stakeholders override public or city interests in the urban ecosystem. The knowledge and resources of the c-CH could be used to satisfy one group’s needs, eliminating other groups or individuals. Disagreements between stakeholders could cause an incapacity of the c-CH and limit its functionality and performance.

Explicit considerations of the ecosystem design pathway, structure and role with identified c-CH raise new scientific questions for further research. On the macro level, concerns include how to consolidate and maintain a competitive dynamic across multiple levels of interactions and different interests of stakeholders. On the micro level, concerns include how authority and political changes have an impact on internal and external stakeholders and their understanding of value proposition and correlation with a need to ensure well-being.

The final results empower future researchers to study this topic in new ways so as to develop a better understanding of how different interests of stakeholders in the urban ecosystem can shape the final outcomes.

## Data Availability

The data presented in this study are available on request from the corresponding author.
